# “We are already person-centred in our practice”—A Qualitative Study of Ambulance Clinicians’ Experiences of Person-Centred Care

**DOI:** 10.3390/healthcare7040115

**Published:** 2019-10-14

**Authors:** Andreas Rantala, Anton Ingoldsson, Eva I Persson

**Affiliations:** 1 Department of Health Sciences, Lund University, SE-221 00 Lund, Sweden; eva_i.persson@med.lu.se; 2 Emergency Department, Helsingborg General Hospital, SE-205 01 Helsingborg, Sweden; anton.ingoldsson@skane.se; 3 Centre of Interprofessional Cooperation within Emergency Care (CICE), Linnaeus University, SE-251 95 Växjö, Sweden

**Keywords:** person-centred care, ambulance services, emergency medical services, qualitative content analysis, ambulance clinicians

## Abstract

The concept of person-centred care (PCC) is considered one of the core competencies in Swedish healthcare. It has increasingly spread and involves treating the patient as a person who is decision-competent and part of the team. The PCC concept has been introduced in the Swedish Ambulance Service setting, but as there has been no previous research on PCC in this context, the aim of the present study was to illuminate ambulance clinicians’ experiences of the introduction of PCC in a Swedish Ambulance Service setting. Data collection took the form of interviews with 15 ambulance clinicians in the southernmost part of Sweden. Qualitative content analysis was employed to analyse the interviews, wherein two categories emerged: organisational perspective and contextual culture. The latent meaning was interpreted as the theme: Seeing the individual in need of care as a person instead of a patient. In conclusion, the concept of PCC was considered a barrier and there was some resistance to its introduction. While PCC enhanced the ambulance clinicians’ stance, e.g., when initiating a caring relationship and encouraging the patient to participate in her/his care, it was also described as a catchphrase that is not applicable to the Ambulance Service as it contributes nothing new to the standard of treatment.

## 1. Introduction

Person-centred care (PCC) has emerged as a core competence in Sweden, where the patient is the target of caring actions, i.e., the patient should be considered a decision-competent person and a natural member of the care team. Thus, clinicians should strive to avoid taking patients’ diseases and diagnoses as their only starting point [1]. PCC in Sweden has mainly been studied in institutional settings and in patients with long-term chronic illnesses [2,3]. To the best of our knowledge, no ambulance service (AS) has introduced PCC as a complete framework, creating an attitude that permeates all its operations. Therefore, the rationale behind this study was to enhance the knowledge of ambulance clinicians’ experiences of PCC in the AS context.

In Sweden, ambulance crews consist of at least one registered nurse together with another registered nurse or emergency medical technician (EMT). Some county councils have increased the requirement, meaning that every ambulance should be crewed by at least one specialist ambulance nurse [4]. Since 2001, there has been a postgraduate programme for specialist ambulance nurses, comprising 60 ECTS (i.e.,40 weeks of full-time study). However, other specialist nurses, e.g., nurse anaesthetists and intensive care nurses, are also common within the Swedish AS [5]. As the AS has evolved and become more advanced, increased focus has been directed towards assessment and decision making [6]. Assessments are primarily conducted through examination of vital signs using various technical aids, and physical examination such as inspection, auscultation and palpation [7]. The ambulance clinicians (ACs) use triage algorithms and treatment guidelines to support decision making [6]. Currently, the main triage system in Sweden is the rapid emergency triage and treatment system (RETTS). The purpose of RETTS is to identify patients in immediate need of medical care and to assess priority levels based on vital signs and the main complaint (emergency symptoms and signs, ESS), where the latter comprises some 45 algorithms, all of which correspond with the ICD-10 classification system [8]. Vital signs and ESS are classified according to a five-tier priority level: red, orange, yellow, green and blue (blue is not applicable in the AS), in descending order of priority and severity levels, where red indicates mortality risk and the need for immediate emergency attention, while green means that the patient can wait for examination. The system provides recommendations on how the patient should be treated regarding the monitoring of vital signs and care process. The different priority levels are linked to a standardized action plan, regardless of the main complaint or availability of resources, i.e., health care personnel in general and physicians in particular [8,9].

The AS is traditionally associated with emergency care, including trauma, resuscitation, accidents and medical conditions that require immediate attention and interventions [10]. However, a previous study reveals that as many as 50% of all assignments are considered non-urgent, i.e., no immediate medical intervention is required. Furthermore, when ACs had arrived at the scene and assessed the patient’s condition only 14% of cases were deemed life-threatening (i.e., requiring transportation to the Accident and Emergency Department with lights and sirens) [11]. As a consequence, concerns have been raised that the Swedish AS requires guidelines that not only focus on medical diseases and emergency conditions, but also include the patient perspective and her/his perceived illness, irrespective of objective vital signs [12]. As argued by Wireklint Sundström and Dahlberg [13], safe decisions are only enabled when objective medical assessment is complemented by a caring assessment.

All threats to a person, such as a disease, illuminate her/his biological and existential vulnerability, but also existential freedom and opportunities. Person-centred ethics means that professionals and patients collaborate: the professionals by providing medical or nursing expertise, while patients contribute their life experience and understanding of what health means to them [14] Thus, the need for patients, significant others and health clinicians to understand and to be understood is considered a central task. Working in accordance with PCC involves caring actions primarily focused on the person that highlight her/his own knowledge and experiences. Furthermore, PCC implies considering both physical and emotional needs in contrast to being solely task-oriented. Shared decision making is also described as a key aspect of PCC [3], and is important for ensuring that the care is experienced as person-centred [15,16]. Consequently, PCC can be understood and concretized based on the three cornerstones of clinical practice developed by Ekman et al. in 2011 [3]: narrative, partnership and documentation. In addition to the traditional medical history, the narrative captures the person’s story about her/his life world and the subjective perspective of illness. [3] This means a shift from a patient as a passive recipient of care consisting of a variety of “what” (e.g., biomarkers) to “who” the person is, in addition to her/his preferences, worries and thoughts [17]. Furthermore, the partnership is created by seeing the person as a genuine and integral part of the care team. This means that people are allowed to participate in the decision making about their own care; thus, self-determination is promoted and codetermination allowed. Finally, documentation of the partnership is a compulsory prerequisite in order to safeguard the outcome of the narrative and decisions taken [3].

On hospital wards where PCC has been implemented, patients experience being listened to and having their own perceptions of the situation recognized. Patients spontaneously articulated that they felt that the health care personnel viewed them as people and did not exclusively focus on their disease. Moreover, it was indicated that not every ailment or characteristic of a patient’s illness had to be addressed or resolved for open listening to be perceived as a positive experience [18]. However, as previously mentioned, in most cases, the ACs’ assessments and decisions are based on the medically oriented RETTS triage system. In addition to objective vital signs, the RETTS has a four question checklist for assessing personal autonomy [9], which might be considered a restrictive and unsatisfactory perspective on the patient. In a majority of all assignments, the ACs only care for one patient at a time, providing exceptional conditions for adopting a person-centred approach in the patient encounter. However, as PCC differs somewhat from the traditional view of care provided by the AS [19], there is need for further elaboration on how PCC is experienced by ACs.

*Aim:* To illuminate ambulance clinicians’ experiences of the introduction of person-centred care in a Swedish Ambulance Service setting.

## 2. Materials and Methods

### 2.1. Design

This study had an inductive design and was conducted by means of interviews in order to probe for experiences in a specific care context where there was no or very little previous knowledge.

### 2.2. Setting

The setting was an ambulance district in the southernmost part of Sweden with six ambulance stations that at the time of the interviews operated eight to 13 land-based ambulances depending on time of day and season. The ambulance district catchment area contained about 300,000 inhabitants in both urban and rural areas, with diversity in terms of socio-economic factors. In 2018, the ambulances in the district handled about 33,000 assignments.

### 2.3. Contextual Introduction of the Person-Centred Care Concept

The AS in the southernmost part of Sweden had received some complaints from patients about negative encounters with the AS in general and ACs in particular. In addition, the county council decided that PCC should be introduced and implemented in all health care organizations. Therefore, in 2016 and 2017, the local ambulance district provided education and training in line with the iPHARIS framework [20] to enhance the competence of all ACs in the interrelated concepts of nursing ethics, professional conduct and PCC. This was conducted through, for example, cathedral lectures and written and oral reflection sessions. Traditionally, the ongoing work-related education (eleven days per year and employee) consisted of medical activities such as cardiac resuscitation, trauma care and technical skills. Hence, PCC was introduced, according to Ekman et al., 2011 [3], as a philosophical and theoretical viewpoint with clinical examples and a shift in power from a paternalistic medical view of the patient to including her/him as a natural member of the team together with the ACs. This introduction to the concept of PCC was considered one of the first steps towards implementing PCC as a complete framework. The management’s intention was that in the near future this approach should constitute a mindset that infiltrates all operations, especially patient–AC encounters. This study was conducted at the same time as the PCC concept was being introduced and reflected upon by the ACs. However, PCC was not considered to be fully implemented.

### 2.4. Sample and Participants

The inclusion criterion for this study was ACs from the local ambulance district in which the PCC concept was introduced. The participants consisted of 15 ACs (10 men/5 women) with a median age of 43 years (range 32–56 years). Two participants were EMTs (18 and 20 years in the AS) and the others were RNs (median 13 years, range 6–30 years in the AS). All but two had training as specialist nurses; six were specialist ambulance nurses, while two were in ongoing specialist training, one was an emergency care nurse, two were intensive care nurses and one was a nurse anaesthetist. The nurses had worked in the AS for <1 year–27 years (median 8 years).

### 2.5. Data Collection

When informed about the study, the ambulance station managers consented to the participation of their ACs. An email was then sent by the managers to all ACs with a request to participate in an interview study. A reminder was also sent. Those who were interested were asked to contact the last author (E.I.P.), who conducted all interviews. The time and place for the interviews was arranged by phone, text or e-mail. One participant wanted the interview conducted in her/his hometown, where a secluded site at the local library was chosen. The other participants wanted to be interviewed at one of the six ambulance stations, where a private area was chosen to minimise the risk of disturbance. The time between the education and the interviews was a minimum of one month and a maximum of six months. All interviews were conducted during December 2017 and January 2018.

The ACs were asked to describe their experiences of how PCC was visible in the care of their patients. Follow-up questions were posed in order to deepen the descriptions of their experiences. Each interview lasted from 30–60 min (median 42 min). The interviews were audio-taped and transcribed verbatim.

### 2.6. Data Analysis

Qualitative content analysis was performed to find the subjective meaning in the interview transcripts [21]. The transcripts were read several times by all three authors to obtain an overall understanding and grasp of both the manifest and latent content [21]. The meaning in every part of the text was then searched for and statements related to the study aim were identified, after which the text was divided into meaning units. The meaning units were condensed and labelled with a code. The various codes were compared based on their differences and similarities and sorted into two categories. Finally, the overall latent content was formulated into one theme. To illustrate the findings, selected quotations were used. Two of the authors (A.I. and E.I.P) conducted the initial analysis independently of each other. Then the results of the analysis were discussed by all authors until consensus was reached. Furthermore, the entire group met several times and discussions were held during different phases of the study.

### 2.7. Ethical Considerations

The ethical code of conduct was followed and conformed to the ethical guidelines adopted by the Swedish Research Council. Consent, confidentiality, utility and information were taken into account in line with the Declaration of Helsinki [22]. As the study did not involve patients or sensitive information, no ethical approval was required according to Swedish ethical protocol and legislation (SFS 2003:460). However, all participants gave their informed written consent and were assigned a number to ensure confidentiality.

## 3. Results

Overall, the participants described the introduction of PCC both as a support and an obstacle. Although not all participants were comfortable with the concept of PCC, they nevertheless acknowledged the importance of making patients feel involved in their care, thus constituting a shift that involved seeing the individual in need of care as a person instead of a patient. Two categories emerged from the analysis (Table 1): *Organisational perspective* and *Contextual Culture*. The latent meaning was interpreted as the theme “seeing the individual in need of care as a person instead of a patient”.

### 3.1. Organisational Perspective 

The organisational perspective was found to consist of sub-categories concerning *Prerequisites for person-centred care in the AS* and *Involvement in development work*, forming the boundaries of and possibilities for PCC in the AS.

#### 3.1.1. Prerequisites for Person-Centred Care in the AS

The AS was considered an appropriate context for working in accordance with the PCC concept, which was described in several different ways. As ACs only care for one patient at a time, it is possible for them to focus on that person. When they are in the patient’s home, they see her/him in context and gain a better picture of the person as a whole, e.g., the housing and social situation that are potentially important pieces of the puzzle of understanding the person’s preferences and how the illness affects her/his everyday life.

*I see the whole person, what they may need, and which different social networks they have and how they get along at home.*

(Informant 13)

There was an awareness that the context is dominated by the medical paradigm. Mandatory clinical guidelines, assessment and measurement instruments and decision support systems all focus on medical issues, constituting a barrier to PCC. However, the participants also emphasized that occasionally they were able to act relatively autonomously, i.e., not necessarily fully adhering to guidelines, in order to optimize the care of the patient. The ACs can themselves choose to prolong the caring session if necessary, without feeling stressed by pending assignments.

*You could stay a bit longer at the scene or in the ambulance arrival zone at the Accident and Emergency Department if you’re not ready or finished … we need to talk a little more with the patient.*

(Informant 13)

#### 3.1.2. Involvement in Development Work

The instructions from management to adopt the concept of PCC could be disturbing and the participants did not feel involved in the process. The ACs who considered that they already worked in accordance with PCC might feel misunderstood. There was a resistance to changing the way they worked, which was especially evident when management and facilitators referred to research on PCC, because it gave the impression that the concept was too inflated. A nurse commented about some of her colleagues:

For example, they already decided before this education that they would not work according to the PCC concept.

(Informant 26)

The participants considered it important that the person-centred way of working in the clinical setting permeated the entire organization, from management level down to the employees. As this was not the case, it resulted in dissatisfaction with managers who were criticized for not seeing the person behind the employee.

*They (managers) do not work in a person-centred way, which reflects on the staff and they do not do so either.*

(Informant 16)

### 3.2. Contextual Culture

The contextual influence in the AS was found to constitute barriers to the use of a person-centred approach in the patient encounter. However, participants also identified PCC as supportive, e.g., by enabling them to find words to express the care that they provide in the AS. Hence, the results concerning culture and organization revealed four sub-categories: *Cultural influence of the medical perspective, Already have Person-Centred Practice, Avoiding Conflicts with Colleagues* and *Professional conduct*. 

#### 3.2.1. Cultural Influence of the Medical Perspective 

After the introduction of PCC, some ACs had a negative view of the concept. Using valuable time to talk about ethics and non-technical issues instead of medical skills was perceived as frustrating. As the medical paradigm was still largely considered to be the dominant framework, some commented that the money would be better spent training the ACs to deal with medical problems instead. However, the participants found that the introduction of the PCC concept was encouraging in terms of shifting the perspective.

*I find it positive that you should try to focus more on patients and relatives than you did before. So you try to break the old approach or trend.*

(Informant 28)

#### 3.2.2. Already have Person-centred Practice

The ACs agreed that they already provided PCC. However, the meaning of working in a person-centred way differed. It could mean making the patient more involved by listening to and involving her/him in decisions about both the symptoms and treatment. It could also mean that the ACs stressed that the patient was also a person when handing her/him over to the nurses at the accident and emergency department. This could manifest itself by, for example, using the patient’s name instead of describing her/him based on a diagnosis or triage assessment prioritization colour. The ACs stated that talking about patients in this way is common among nurses at the accident and emergency departments.

*I am not bringing a yellow abdominal pain, I am actually bringing a person.*

(Informant 13)

Furthermore, the participants described that working in a person-centred way involved engaging in activities that demanded "that little bit extra", e.g., activities that took extra time. It was also described as being about taking more care of patients with psychosocial issues.

*…those who, e.g., have depression or anxiety, you need to talk to them and come to a mutual agreement.*

(Informant 26)

When working according to the PCC concept, the participants felt that it was important not to blame the patient for summoning an ambulance. However, some participants felt that PCC was a popular new concept for something they were already doing. It almost became somewhat frustrating having to learn about something that they already knew and practiced. Thus, the participants reported that ACs sometimes mocked the concept of PCC because they felt they had been ordered to employ it. However, it was also a joke between colleagues when in addition to the medical treatment one made a little extra effort for the patient.

*You clap each other on the shoulder and say, “that was person-centred care”.*

(Informant 26)

#### 3.2.3. Avoiding Conflicts with Colleagues

The AS care culture was described as quite tough due to its tradition of emergency assignments involving life and death situations. Hence, breaching this culture and tradition to talk about PCC could imply conflicts with colleagues. There was a feeling of discomfort when discussing patient situations that were considered to be not person-centred, especially in relation to colleagues who were negative about PCC as a concept.

*It´s delicate to raise… maybe I think this wasn´t all right. It´s really hard to raise the subject with your colleague with whom you work closely together.*

(Informant 26)

Some participants reported the risk of being seen as a wimp in a context where a fairly tough attitude was expected. The participants stated that care within the AS concerns quick decisions in response to vital signs, possibly a matter of life and death. Hence, although embracing the PCC concept, ACs tend to advocate the medical perspective, which prevents them from performing and discussing PCC.

*It´s regarded as foolish to say that you work in a PCC way… many won´t admit thinking like that, although they do so.*

(Informant 14)

#### 3.2.4. Professional Conduct

The participants described that some ACs were more likely to end up in conflict with patients and relatives and often questioned what was to be done at the scene, i.e., if the assignment was considered urgent or not from the AC perspective. There was a view among the participants that suitability for the AS was mainly a result of one’s personality. Hence, it was perceived that some people can care for patients, while others are unable to do so, irrespective of training, which further strengthened the negative opinions about the time spent on learning and reflecting on the PCC concept.

You can’t sit and learn how to take care of people, you must be the type who has that ability. But many want to work in such an acute area and it’s a bit cheeky.

(Informant 29)

There were many descriptions of how the ACs reflected on the care they provided to a patient, including not only the acute medical perspective, but also the treatment and the relationship with the patient and/or her/his significant other.

*…perhaps the conclusion is ok, here we did not reach out to the patient and could have done something differently. We then try to reflect on the situation and what we should have done.*

(Informant 27)

The concept of PCC could also lead to a discussion between colleagues, especially in the case of non-conveyance of the patient. In such situations, ACs questioned themselves about whether the care provided was really in accordance with PCC. Those who were positive about the education and introduction of PCC realised that these discussions could lead to reflection on their own behaviour. The beginning of an awareness that changes need time could then be seen.

*This is where we must begin to think about our own behaviour.*

(Informant 16)

In general, there was a strong awareness that as an AC, one was in a position of power in relation to the patient. The participants stated that this was discussed and reflected on between colleagues. There was also an awareness that entering the patient’s home as an AC and wearing a uniform could possibly make the patient feel inferior. Those who reflected on their position of power described how they tried to make the patient feel comfortable by listening and asking questions to make sure that she/he felt involved.

*If we do not care about what the patient says it is like a product that is to be processed at the factory. You use your role as a position of power, as it in fact is.*

(Informant 26)

However, some participants were not aware of their position of power, and thus relinquished responsibility to the patient and significant others.

*Yes, but much depends on how receptive the patient and significant others are to information.*

(Informant 18)

## 4. Discussion

This study reported on ACs’ experiences of PCC following the introduction of the concept in a prehospital context. The participants’ experiences of PCC were widely spread across the entire spectrum. The concept of PCC was considered to be both a barrier and support to working in a person-centred manner in clinical practice. On the one hand, PCC enabled the ACs to find the right words: to, e.g., initiate a caring relationship in order to encourage the patient to participate in the care and be a partner in the decision-making process. On the other hand, PCC was described as a catchphrase that was not applicable to the AS, as it contributed nothing new or unique to the quality of assessment or treatment. This was congruent with Naldemirci, et al. [23], who found conflicting and divergent views on PCC and that some practitioners did not comprehend that PCC could and should change their everyday practice. As currently and historically the AS has been weighed down by a legacy based on a biomedical paradigm [6], introducing PCC could be challenging, as it is an abstract concept based on philosophies that must be interpreted and adopted in concrete practice [23]. Care built on a biomedical paradigm can be rigid and opposed to change, e.g., the application of different policies. PCC restricts and challenges clinicians’ routines and goals [24,25]. The results indicate that some ACs thought that it was a waste of time talking about ethics and non-technical medical skills, and that the money would be better spent on training ACs to deal with medical problems instead. This has also been shown in ambulance nurse training programmes, where a review of curricula revealed a greater focus on medical knowledge than on nursing/caring science [26]. Furthermore, a Swedish study on ambulance nurses showed that they adopted either a medical or a caring perspective depending on the condition of the patient. The ambulance nurses considered that establishing an interpersonal caring relationship had low priority in urgent and emergency situations. Hence, interpersonal and medical aspects were not perceived to be equally significant [27].

The participants in this study found that the basis for implementing and working in accordance with the cornerstones of the PCC concept in the AS could be very advantageous, particularly as the context makes it possible to focus on one patient at a time and collect information about her/his home situation. Focusing on the encounter is paramount as, from a patient perspective, being a person in need of care provided by the AS means being vulnerable, exposed and feeling powerless [28,29,30]. Bearing the patient perspective in mind, ACs should attempt to capture the most important dimensions of suffering and to use all their senses to assess and try to understand what caused the patient’s request for help [31]. The results indicate possible barriers to future PCC implementation and practice, as is often the case when changes are introduced [32]. One of the reasons for resistance to the introduction of PCC described in our study was that the ACs did not feel involved in the process. The decision had come from management, and ACs who already worked according to PCC could feel ignored and misunderstood. Another obstacle when introducing the concept of PCC was the fact that this approach did not permeate the entire organization. The managers were criticized for not seeing the person, i.e., the AC, behind the employee. This was congruent with other studies in different contexts. When developing the quality of care, it is vital that managers are aware of and interested in the work of their staff [25,33,34]. An important implication from our results is that leaders should serve as role models [25]. In a recent Swedish study, managers in the AS who had a background as either a nurse or physician were interviewed about their perspective on care. Physician managers added the importance of nursing knowledge to the medical perspective. However, nurse managers, who were traditionally closer to the day-to-day clinical operations, only mentioned the medical perspective [35], thus neglecting important subjective viewpoints. This may constitute a major obstacle to the integration of a person-centred perspective on both patients and employees.

The participants in this study reported the risk of being seen as a wimp when trying to discuss person-centred issues, as the AS context was characterised as rather tough and medically orientated. A study indicated that ACs find it difficult to raise the issue of bad behaviour on the part of colleagues [36]. The workplace culture can therefore hinder discussions about behaviours in the encounter between colleagues, as well as concerning patients.

The findings suggest that the participants were aware of their position of power in the encounter with the patient, and possibly her/his significant others. From the patients’ perspective, Kristensson Uggla [24] proposed that a patient entering the healthcare system tends to be automatically placed at a triple disadvantage, consisting of a institutional disadvantage based on the hierarchical healthcare regime, an existential disadvantage that concerns the person’s perceived illness and how it affects everyday life and a cognitive disadvantage, possibly involving a knowledge gap. This triple disadvantage illuminates the risk of asymmetry in the relationship between the healthcare professional and the person in need of care [37]. Thus, it is important to bear in mind that ACs have the power to interpret symptoms and signs, diagnose and ultimately to decide on examinations, care, treatment and interventions. Encouraging patients to participate in their care and enabling them to become a partner is paramount for balancing this asymmetrical relationship [38]. The ACs experienced that they already had a person-centred approach in clinical practice, and thus expressed that the introduction of PCC contributed no new knowledge as they already worked in that manner. Similar results were found in a study conducted in a surgical hospital setting, where an intervention employing a person-centred communication approach was performed. However, most professionals thought that they already spoke to patients in this way before the intervention, as it was considered self-evident [39].

In today’s healthcare system, the PCC concept has had a well-deserved major impact, and policy decisions have been made at various levels that care should be person-centred. Therefore, no organisation, the AS included, can refuse to acknowledge PCC, but does this mean that the care provided is actually person-centred in reality and thus PCC is truly implemented? Consequently, in accordance with Santana et al. [40], future research on the implementation of PCC within the AS should further explore patients’ experiences as well as investigating the process, i.e., cultivating communication, respectful, compassionate care and involving patients in their care and outcome, e.g., accessibility of care and patient reported experience measures.

Trustworthiness was ensured as far as possible throughout the entire study process. To increase credibility [41] and to enrich the variation in the phenomenon, both female and male nurses and EMTs were recruited, providing a large spread in age and number of years’ experience within the AS. ACs had different specialist training, with the exception of two who had no specialist training [41]. The introduction of the PCC concept was conducted in one ambulance district, but six ambulance stations were involved. There were only 15 informants, which could be considered a few. However, there was variation in the phenomenon, and the final interviews did not add anything new. It cannot be ruled out that further variation could be achieved if more participants were included.

To ensure the objectivity of the interviews and that the results were credible, all interviews were conducted by the last author (E.I.P.), who was the only one with no connection to the AS. The analysis was conducted by the entire research group, who met several times, and discussions were held during different phases of the study [21]. In addition, the interviews were conducted for a relatively short period, December 2017 to January 2018, which reduced the risk of organizational changes being implemented. Furthermore, to strengthen transferability [41], the study provided a clear description of the participants’ characteristics, data collection and the analysis process. Additionally, to further strengthen the study’s credibility, the results are illustrated by quotation [42]. Generalisation may be limited by the fact that only one organisation was involved.

## 5. Conclusions

The findings indicate that from the perspective of ACs, PCC can be described from organizational and cultural perspectives. The concept of PCC was considered to be both a barrier and a support to working in a person-centred manner in clinical practice. On the one hand, PCC enables ACs to find the right words, e.g., to initiate a caring relationship in order to encourage the patient to participate in their own care and to be a partner in the decision-making process. On the other hand, PCC is described as a catchphrase that is not applicable to the AS, because it contributes nothing new or unique to the standard assessment or treatment. Overall, the participants found that person-centredness represented a shift from viewing the individual in need of care as a patient to seeing her/him as a person.

The AS organisation traditionally comprises a context with a biomedical bias; triage algorithms, treatment guidelines and educational efforts rarely widen the perspective of viewing a patient as a person. In that sense, the AS counteracts the implementation of a person-centred approach. No organisation can ignore the need to be a person-centred healthcare provider. However, within the AS, the contextual culture influences the organisation as the medical paradigm is emphasized. Therefore, the AS must adopt a person-centred approach, thus including the patient as part of the team by means of implementing PCC in practice, not just as empty words without any content.

## Figures and Tables

**Table 1:** Summary of the theme, categories and sub-categories.

Seeing the Individual in Need of Care as a Person Instead of a Patient	
**Organisational perspective**	Contextual culture
Prerequisites for Person-Centred Care in the AS Involvement in Development Work	Cultural influence of the medical perspective Already have person-centred practice Avoiding conflicts with colleagues Professional conduct

## References

[1] Edberg A., Ehrenberg A., Friberg F., Wallin L., Wijk H. (2013). Omvårdnad på Avancerad Nivå-Kärnkompetenser Inom Sjuksköterskans Specialistområden (Nursing at Advanced Level-Core Competences in the Nurse Specialist Areas).

[2] The Swedish Society of Nursing, Swedish Society of Medicine, The Swedish Association of Clinical Dietitians (2019). Personcentrerad vård—en kärnkompetens för god och säker vård [Person-centred care—a core competence for good and safe care].

[3] Ekman I., Swedberg K., Taft C., Lindseth A., Norberg A., Brink E., Carlsson J., Dahlin-Ivanoff S., Johansson I.L., Kjellgren K. (2011). Person-centered care-ready for prime time. Eur. J. Cardiovasc. Nurs..

[4] Suserud B.-O. (2005). A new profession in the pre-hospital care field--the ambulance nurse. Nurs. Crit. Care.

[5] RAS. Riksföreningen för ambulanssjuksköterskor (2012). Riksföreningen för ambulanssjuksköterskor. Kompetensbeskrivning Legitimerad Sjuksköterska Med Specialistsjuksköterskeexamen Med Inriktning Ambulanssjukvård (Competence Description Registered Nurse with Specialist Nursing Pre-hospital Emergency Nursing).

[6] Bremer A., Suserud B., Lundberg L. (2016). Dagens ambulanssjukvård (The ambulance care of today). Prehospital Akutsjukvård (Prehospital Emergency Care).

[7] Nyström M., Herlitz J., Suserud B., Lundberg L. (2016). Möte mellan två kunskapsområden (Meeting between two areas of knowledge). Prehospital akutsjukvård (Prehospital Emergency Care).

[8] Widgren B., Jourak M. (2011). Medical Emergency Triage and Treatment System (METTS): A new protocol in primary triage and secondary priority decision in emergency medicine. J. Emerg. Med..

[9] Widgren B. (2012). RETTS akutsjukvård direkt (RETTS Emergency Care Directly).

[10] Gårdelöv B., Suserud B., Lundberg L. (2009). Ambulanssjukvårdens utveckling i Sverige (Development of the Prehospital Emergency Care in Sweden). Prehospital Akutsjukvård (Prehospital em;ergency c;are).

[11] Ek B., Edström P., Toutin A., Svedlund M. (2013). Reliability of a Swedish pre-hospital dispatch system in prioritizing patients. Int. Emerg. Nurs..

[12] Lederman J., Svensson A., Rantala A. (2018). Absence of evidence-based and person-centred guidelines in the Swedish Emergency Medical Services: A patient safety issue?. Int. Emerg. Nurs..

[13] Wireklint Sundström B., Dahlberg K. (2011). Caring assessment in the Swedish ambulance services relieves suffering and enables safe decisions. Int. Emerg. Nurs..

[14] Ekman I., Hedman H., Swedberg K., Wallengren C. (2015). Swedish initiative on person centred care. BMJ.

[15] DiLollo A., Favreau C. (2010). Person-centered care and speech and language therapy. Semin. Speech Lang..

[16] McCormack B., McCance T. (2010). Person-Centered Nursing: Theory and Practice.

[17] Ricoeur P. (1992). Oneself as Another.

[18] Alharbi T.S., Carlström E., Ekman I., Jarneborn A., Olsson L.E. (2014). Experiences of person-centred care—Patients’ perceptions: Qualitative study. BMC Nurs..

[19] Rosen H., Persson J., Rantala A., Behm L. (2018). A call for a clear assignment-A focus group study of the ambulance service in Sweden, as experienced by present and former employees. Int. Emerg. Nurs..

[20] Harvey G., Kitson A. (2015). Implementing Evidence-Based Practice in Healthcare: A Facilitation Guide.

[21] Graneheim U.H., Lundman B. (2004). Qualitative content analysis in nursing research: Concepts, procedures and measures to achieve trustworthiness. Nurse Educ. Today.

[22] World Medical Association (2013). World Medical Association Declaration of Helsinki-Ethical Principles for Medical Research Involving Human Subjects. JAMA.

[23] Naldemirci O., Wolf A., Elam M., Lydahl D., Moore L., Britten N. (2017). Deliberate and emergent strategies for implementing person-centred care: A qualitative interview study with researchers, professionals and patients. BMC Health Serv. Res..

[24] Kristensson Uggla B., Ekman I. (2014). Personfilosofi-filosofiska utgångspunkter för personcentrering inom hälso-och sjukvård (Personal Philosophy-filosophical starting points for person-centeredness in health care). Person-Centeredness in Health Care. From Philosophy to Practice (Personcentrering Inom Hälso-Och Sjukvård. Från Filosofi Till Praktik).

[25] Moore L., Britten N., Lydahl D., Naldemirci O., Elam M., Wolf A. (2017). Barriers and facilitators to the implementation of person-centred care in different healthcare contexts. Scand. J. Caring Sci..

[26] Sjölin H., Lindstrom V., Hult H., Ringsted C., Kurland L. (2015). What an ambulance nurse needs to know: A content analysis of curricula in the specialist nursing programme in prehospital emergency care. Int. Emerg. Nurs..

[27] Svensson C., Bremer A., Holmberg M. (2019). Ambulance nurses experiences of patient relationships in urgent and emergency situations: A qualitative exploration. Clin. Ethics.

[28] Bremer A., Dahlberg K., Sandman L. (2009). To survive out-of-hospital cardiac arrest: A search for meaning and coherence. Qual. Health Res..

[29] Elmqvist C., Fridlund B., Ekebergh M. (2008). More than medical treatment: The patient’s first encounter with prehospital emergency care. Int. Emerg. Nurs..

[30] Holmberg M., Forslund K., Wahlberg A., Fagerberg I. (2014). To surrender in dependence of another: The relationship with the ambulance clinicians as experienced by patients. Scand. J. Caring Sci..

[31] Bremer A., Dahlberg K., Sandman L. (2012). Balancing between closeness and distance: Emergency medical services personnel’s experiences of caring for families at out-of-hospital cardiac arrest and sudden death. Prehosp. Disaster Med..

[32] Johnson M., O’Hara R., Hirst E., Weyman A., Turner J., Mason S., Quinn T., Shewan J., Siriwardena A.N. (2017). Multiple triangulation and collaborative research using qualitative methods to explore decision making in pre-hospital emergency care. BMC Med. Res. Methodol..

[33] Bergh A.L., Karlsson J., Persson E., Friberg F. (2012). Registered nurses’ perceptions of conditions for patient education-focusing on organisational, environmental and professional cooperation aspects. J. Nurs. Manag..

[34] Bokberg C., Behm L., Wallerstedt B., Ahlstrom G. (2019). Evaluation of person-centeredness in nursing homes after a palliative care intervention: Pre-and post-test experimental design. BMC Palliat. Care.

[35] Tegelberg A., Jangland E., Juhlin C., Muntlin Athlin A. (2019). Who is in charge of the care of patients with acute abdominal pain? An interview study with managers across the acute care chain. J. Clin. Nurs..

[36] Williams B., Beovich B., Flemming G., Donovan G., Patrick I. (2017). Exploration of difficult conversations among Australian paramedics. Nurs. Health Sci..

[37] Ekman I. (2014). Personcentrering Inom Hälso-Och Sjukvården. Från Filosofi Till Praktik (Person-Centeredness in Health Care. From Philosophy to Practice).

[38] Bremer A., Suserud B., Lundberg L. (2016). Den Mångfacetterade Delaktigheten (The multifaceted participation). Prehospital Akutsjukvård (Prehospital Emergency Care).

[39] Friberg F., Wallengren C., Håkanson C., Carlsson E., Smith F., Pettersson M., Kenne Sarenmalm E., Sawatzky R., Ohlen J. (2018). Exploration of dynamics in a complex person-centred intervention process based on health professionals perspectives. BMC Health Serv. Res..

[40] Santana M.J., Manalili K., Jolley R.J., Zelinsky S., Quan H., Lu M. (2018). How to practice person-centred care: A conceptual framework. Health Expect..

[41] Lincoln Y.S., Guba E.G. (1985). Naturalistic Inquiry.

[42] Polit D.F., Beck C.T. (2008). Nursing Research: Generating and Assessing Evidence for Nursing Practice.

